# Alectinib combined with 5-aminolevulinic acid-based photodynamic therapy promotes apoptosis in endometrial cancer cells by targeting ferrochelatase

**DOI:** 10.3389/fonc.2026.1800668

**Published:** 2026-06-30

**Authors:** Pengfei Wang, Xian Zhang, Haijing Li, Lujiadai Xue, Yajuan Deng, Hu Li, Kunyong Xie, Xiaoyu Wang, Yuanhong Chen

**Affiliations:** 1 Department of Gynecology, Tianhe District, The First Affiliated Hospital of Jinan University, Guangzhou, China; 2 Department of Gynecology, The Sixth Affiliated Hospital of Jinan University (Dongguan Eastern Central Hospital), Dongguan, Guangdong, China; 3 Department of Gynecology, The Affiliated Shunde Hospital of Jinan University, Foshan, Guangdong, China

**Keywords:** 5-aminolevulinic acid, alectinib, endometrial cancer, photodynamic therapy, protoporphyrin-IX

## Abstract

**Background:**

Hysterectomy is the standard treatment for early-stage endometrial cancer, but this type of surgery is associated with corresponding surgical trauma. Ferrochelatase (*FECH*) catalyzes the conversion of protoporphyrin IX (PpIX) to inert heme, thereby limiting photodynamic therapy (PDT) efficacy. Alectinib (an ALK inhibitor) has been shown to suppress *FECH* activity via off-target effects. Nevertheless, whether alectinib (ALE) can regulate PDT efficacy in endometrial cancer by targeting *FECH* remains to be elucidated.

**Objective:**

To investigate the antitumor effect and underlying mechanism of alectinib combined with 5-aminolevulinic acid-based photodynamic therapy (ALA-PDT) on endometrial cancer cells via targeting *FECH*.

**Design:**

An *in vitro* experimental study was conducted on human endometrial cancer Ishikawa (ISK) and HEC-1-A cell lines. The cytotoxicity of alectinib was detected by CCK8 assay to screen the optimal concentration. Confocal laser microscopy was used to observe the subcellular localization of PpIX. Intracellular Reactive Oxygen Species (ROS) generation was detected by fluorescent probe staining, and cellular heme content was determined using a commercial heme assay kit. qPCR and Western blotting were performed to measure the mRNA and protein expression levels of *FECH* after alectinib treatment. Annexin V-AF647/propidium iodide (PI) staining combined with flow cytometry was applied to quantify the apoptotic rate of endometrial cancer cells following LED illumination after single or combined treatment with alectinib and ALA.

**Results:**

Using ISK and HEC-1-A cells, we found alectinib’s IC_50_ values were 38.83 μM and 42.70 μM, respectively. Confocal microscopy showed that PpIX co-localized with mitochondria, and alectinib and 5-aminolevulinic acid (ALA) groups exhibited higher red fluorescence intensity than the control. Compared with ALA-PDT alone, combination treatment with alectinib further promoted intracellular ROS accumulation and significantly reduced cellular heme levels. Alectinib treatment significantly increased *FECH* mRNA and protein expression (P<0.05). Flow cytometry results indicated that combined alectinib and ALA treatment, followed by LED illumination, induced a significantly higher apoptosis rate than ALA alone or alectinib alone.

**Conclusions:**

Alectinib targets *FECH* activity to promote mitochondrial enrichment of PpIX, and its combination with ALA-PDT exerts potent antitumor effects via enhanced cell apoptosis.

## 1 Introduction

Endometrial cancer is the sixth most common cancer in women, and its incidence is gradually increasing, with a trend toward younger age (1). Hysterectomy is a main treatment for early-stage endometrial cancer in current guidelines, but this procedure inevitably results in corresponding surgical trauma (2). As the most widely used conservative treatment, progesterone therapy relies on high-dose progesterone to inhibit the proliferation of tumor cells in progesterone receptor-positive patients. However, its response rate is only 60%-70% in early low-grade EC, and the recurrence rate is as high as 30%-40% after drug withdrawal (3). PDT is based on the selective accumulation of photosensitizers in tumor tissues and the generation of ROS by specific wavelength light to kill tumors (4). When light energy is absorbed, electrons in PpIX molecules transition from the ground state to the excited state and can transfer this energy to molecular oxygen to generate ROS, which is responsible for cell oxidation and tumor cell necrosis/apoptosis (5). *FECH* catalyzes the synthesis of heme B by inserting iron into PpIX (6). Heme B lacks photosensitive activity, which reduces the efficacy of PDT. Down-regulation of *FECH* activity can increase the production of PpIX (7). At present, most studies at home and abroad use exogenous ALA to promote PpIX synthesis in tumor tissues (8). A high ALA dose well above physiological levels is often required for sufficient PpIX accumulation and tumor suppression, which may induce dose-dependent cutaneous phototoxicity and potential neurotoxicity, thereby limiting clinical applicability (9). Induction of PpIX accumulation by knockdown of *FECH* gene expression by siRNA was established in previous studies (10). However, the *in vivo* delivery and targeting efficiency of siRNA remain limited, hindering its clinical translational application (11). Therefore, identifying a small-molecule *FECH* inhibitor with favorable pharmacokinetic properties could provide a clinically viable alternative.

**Figure 1:**
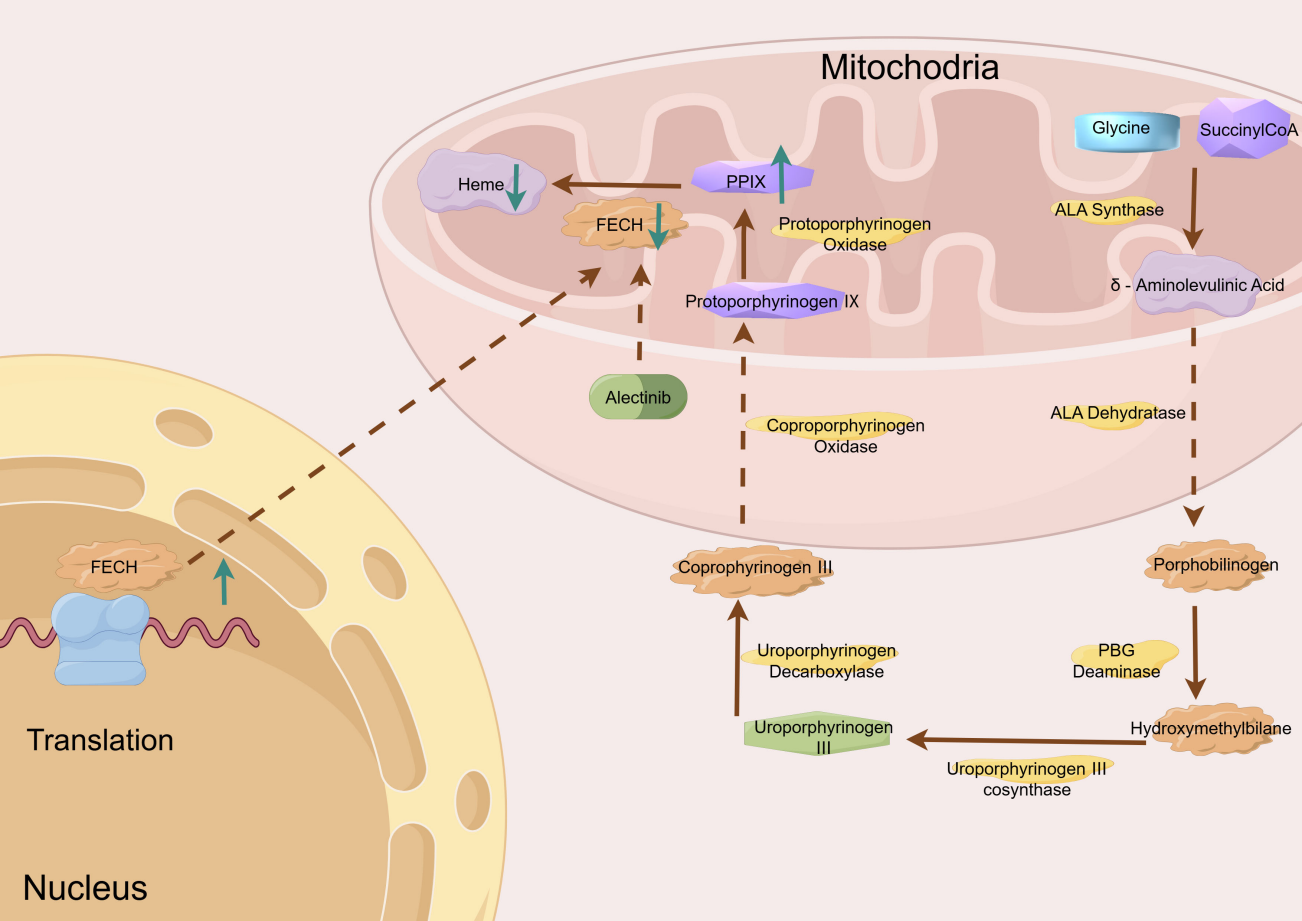
Schematic of the mechanism of PpIX metabolism in tumor cells and induction of PpIX accumulation by inhibition of *FECH*.

Alectinib, a second-generation ALK tyrosine kinase inhibitor, has shown remarkable efficacy in the treatment of ALK-positive non-small cell lung cancer (NSCLC) (12). Recent studies have found that alectinib may interfere with the heme metabolic pathway through off-target effects and inhibit the activity of *FECH* (13). This finding provides a new direction for the enhancement of PDT: using the inhibitory effect of alectinib on *FECH* is expected to enhance the selective killing ability of ALA-PDT on tumor cells. Nevertheless, the regulatory mechanism of alectinib on the *FECH*-PpIX axis, as well as its regulatory role in ALA-PDT against endometrial cancer, has not yet been reported, and the relevant molecular mechanism remains to be systematically explored. Therefore, this study proposes to repurpose alectinib as an *FECH* inhibitor to elevate specific PpIX accumulation in tumor tissues. Combined with the ROS-mediated cytotoxicity of ALA-PDT, this study aims to establish a novel selective therapeutic strategy for EC (**Figure 1**).

## 2 Materials and methods

### 2.1 Cell culture

Human endometrial cancer cells HEC-1-A and ISK were purchased from Wuhan Zishan Biotechnology Co., Ltd. (Wuhan, China). Cells were cultured in high-glucose DMEM without phenol red (Gibco, Cat. #: 31053028) supplemented with 10% fetal bovine serum (Gibco, Cat. #: 10099141C), 100 U/mL penicillin, and 100 μg/mL streptomycin (Gibco, Cat. #: 15140122). Cells were cultured in a 37 °C incubator with 5% CO_2_. Cells in the logarithmic growth phase were routinely passaged every three days using 0.25% trypsin-EDTA solution (Gibco, Cat. #: 15400054). All human cell lines were authenticated via STR profiling within the past three years, and all experiments were conducted using mycoplasma-free cells.

### 2.2 CCK8 cytotoxicity assay

The cell concentration was adjusted to 5×10^3^ cells/mL, and 100 μL cell suspension was added to each well of the 96-well plate. After cell adhesion, alectinib (MedChemExpress, Cat. #: HY-13011) was diluted in serum-free medium into different concentration gradients (ranging from 0.5 to 100 μM with concentrations of 0.5, 1, 2, 5, 10, 20, 50, and 100 μM). At the same time, a blank control group and a cell control group were set up. 100 μL of different concentrations of drug solution was added to each well. The culture was continued for 24 hours. The CCK8 reagent (Beyotime, Cat. #: C0038) was mixed with serum-free medium at a 1:10 ratio. 100 μL of CCK8 mixture was added to each well. The absorbance (OD) at 450 nm was measured by a microplate reader.

### 2.3 Cell grouping and seeding

Endometrial cancer cells were seeded in six-well plates at a density of 2.0×10^5^ cells per well. Cells were divided into four main experimental groups: NC, ALA alone (MedChemExpress, Cat. #: HY-N0305R), ALE alone, and the combined ALA+ALE group. Gradient concentrations of ALA (2, 20, 100 μM) and alectinib (5, 10, 20 μM) were set for subsequent screening, with 20 μM ALA and 20 μM alectinib selected as the optimal concentrations for formal combined experiments. Drugs were added to the medium, and all plates were wrapped with tin foil to avoid light exposure.

### 2.4 Fluorescence imaging by laser confocal microscope

After 24 hours of continuous culture, the cells were double-labeled with mitochondrial-specific staining and nuclear staining, and then observed by confocal laser scanning fluorescence microscopy (Leica STELLARIS 5). MitoTracker Green was used for mitochondrial labeling with excitation/emission wavelengths of 490 nm/516 nm. Nuclei were stained with Hoechst 33342 at 350 nm (excitation) and 461 nm (emission). Intracellular PpIX fluorescence was detected at excitation of 545/30 nm and emission of 620/60 nm. Pictures were randomly selected to measure the fluorescence intensity of cells. Quantitative analysis of laser confocal images was performed on ImageJ software, and the cell area and average fluorescence intensity were measured.

### 2.5 ROS detection

Intracellular ROS levels were detected using a DCFH-DA ROS detection kit (Abbkine, Cat. #: KTB1910). After 24 h of drug treatment and LED light illumination (as described in Section 2.7), cells were washed twice with pre-cooled PBS, then incubated with 0.5 mL (12-well plate) DCFH-DA working solution (1:1000 diluted in serum-free DMEM) at 37 °C in the dark for 20 min. After washing with PBS to remove unbound DCFH-DA, cells were observed and imaged under a fluorescence microscope (Leica STELLARIS 5). DCF fluorescence (reflecting ROS levels) was detected with excitation wavelength 488 nm and emission wavelength 525 nm. Light negative and blank control groups were included to exclude interference.

### 2.6 Heme detection

Intracellular heme content was determined using a heme assay kit (LABLEAD, Cat. #: YTXH200) according to the manufacturer’s protocol. After 24 h of drug treatment, cells were collected, washed twice with cold PBS, and lysed on ice for 30 min (vortexed every 10 min). Lysates were centrifuged at 14000 rpm for 10 min at 4 °C, and supernatants were collected. 50 μL supernatant and 200 μL reaction solution were added to 96-well plates, incubated at room temperature for 5 min, and absorbance at 400 nm was measured. Heme concentration was calculated based on the absorbance values of blank and calibrator samples following the kit instructions.

### 2.7 Quantitative polymerase chain reaction analysis

Cells were divided into solvent control (0.1% DMSO) and alectinib-treated groups. After 24 h of treatment, total RNA was extracted using TRIzol reagent (Ambion, Cat. No. 15596018) and reverse transcribed into cDNA for subsequent detection of *FECH* mRNA expression. *β-actin* was used as the internal reference gene. The primer sequences were listed as follows: *FECH*: forward 5′-AGGAAGCCGAAAACTGGAAT-3′, reverse 5′-TGCGGTACTGCTCTTGAATC-3′; *β-actin*: forward 5′-GGCATCGTGATGGACTCCG-3′, reverse 5′-GCTGGAAGGTGGACAGCGA-3′. The 2^-ΔΔCt^ method was used to calculate the relative expression of target genes. All primer pairs were validated to ensure specific amplification without primer-dimer formation or non-specific products.

### 2.8 Western blot analysis

Cells were harvested 48 h after alectinib treatment and lysed on ice with RIPA buffer supplemented with protease inhibitors. Cell lysates were centrifuged, and the supernatant containing total protein was collected. Protein concentration was quantified using a BCA assay, and samples were normalized to equal protein levels. Equal amounts of protein were mixed with loading buffer and denatured by boiling. Proteins were separated by SDS-PAGE and then wet-transferred onto PVDF membranes. Membranes were blocked with 5% non-fat milk at room temperature for 1–2 h, followed by overnight incubation with primary antibody against *FECH* (Invitrogen, Cat. No. PA5-106355) at 4 °C. After washing with TBST, membranes were incubated with HRP-conjugated secondary antibody (Abmart, Cat. No. M21003) for 1 h at room temperature. Protein bands were visualized using ECL chemiluminescence reagent. The gray values of target proteins and internal controls were quantified with ImageJ software to calculate relative protein expression levels.

### 2.9 LED light source illumination

After 24 h of drug incubation, the culture medium was replaced with fresh phenol red-free medium before light irradiation. The light negative control group was set up at the same time. A customized narrow-band low-power LED light source was used with the following optical parameters: center wavelength 405 nm, FWHM 35 nm, and the output mode is non-focused direct light. The wavelength of the light source is closely matched with the characteristic absorption spectrum of PpIX, which can efficiently excite the photosensitizer to produce ROS. For experiments, the vertical distance between the light source and the 6-well cell culture plate was fixed at 20 cm, and the irradiation intensity was calibrated to 6 mW/cm² at the cell growth plane (bottom of the culture wells) by an optical power meter. The irradiation time of this experiment was 20 minutes (1200 seconds), so the total light dose was 6 mW/cm² × 1200 s = 7.2 J/cm². At the same time, the low-power design avoids non-specific thermal damage. Subsequently, flow cytometry was performed to detect relevant cellular indicators.

### 2.10 Flow cytometry analysis

Following a 24-hour treatment period, cells were stained with Annexin V-AF647 (Goonie, Cat. No.: 100-102) to assess phosphatidylserine externalization and co-stained with PI to differentiate between early apoptotic and necrotic cells. The apoptotic profile was subsequently analyzed by flow cytometry. The combined percentage of early and late apoptotic cells corresponds to the overall apoptotic rate.

### 2.11 Statistical analysis

All experimental data are presented as mean ± standard deviation (SD). GraphPad Prism 9.5.0 software was used for statistical analysis. Each group was set up in triplicate, and all experiments were repeated three times independently. Student’s t-test was used for comparisons between two groups. For comparisons among three or more groups, one-way ANOVA was performed first. A *post hoc* Dunnett’s test was adopted when each experimental group was compared with the control group. The Games-Howell test was applied in cases of unequal variance or unequal sample size. If the one-way ANOVA result was not statistically significant (p ≥ 0.05), no *post hoc* tests were performed. A value of p < 0.05 was considered statistically significant. *p < 0.05, **p < 0.01, ***p < 0.001, ****p < 0.0001.

## 3 Results

### 3.1 Screening of optimal alectinib concentrations by CCK8 assay

**Figure 2:**
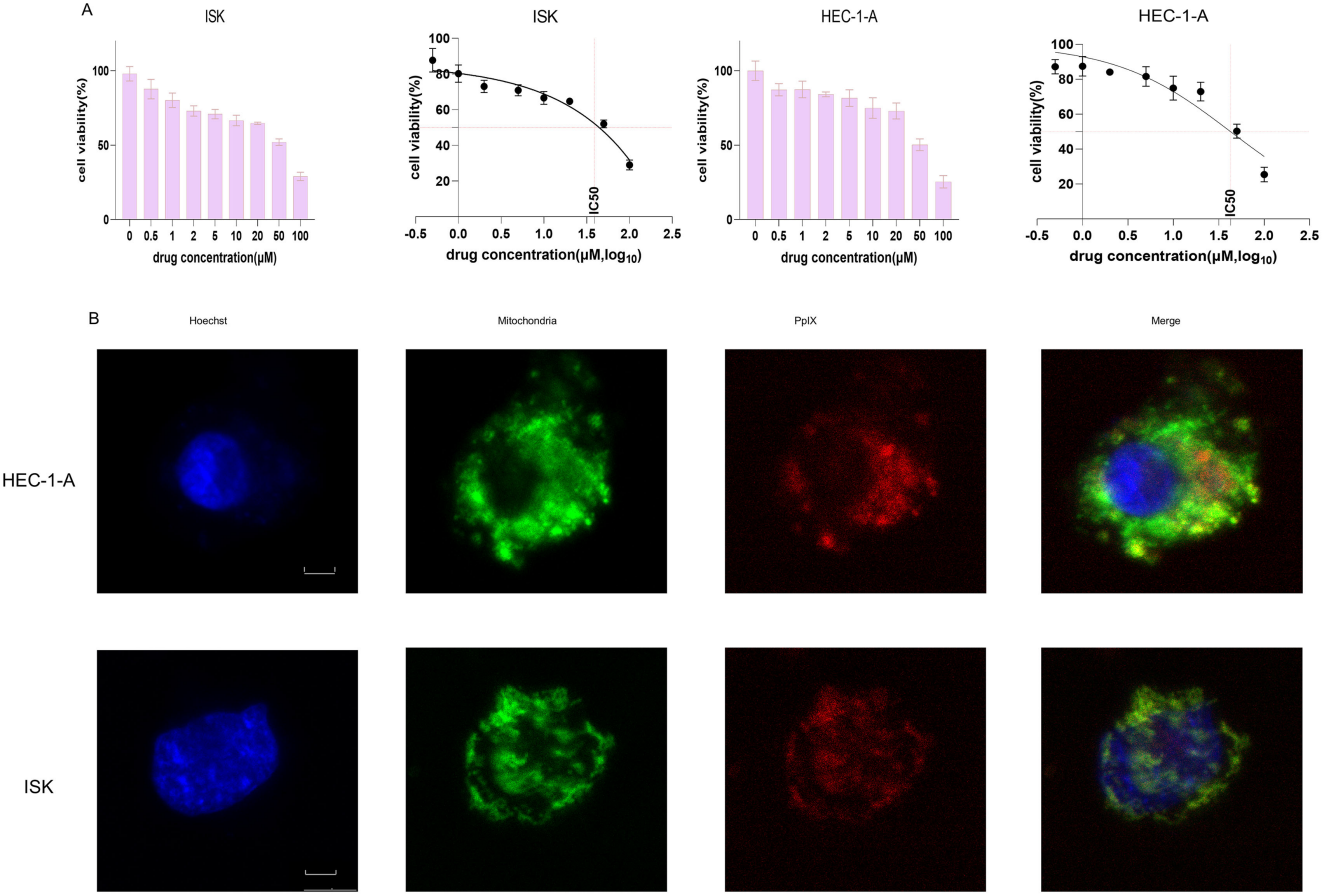
**(A)** CCK8 assay was used to detect the toxicity of alectinib on endometrial cancer cells. **(B)** Confocal fluorescence localization of two endometrial cancer cell lines after the use of alectinib.

The CCK8 assay was used to detect the toxic effect of alectinib on endometrial cancer cells to clarify the concentration range of alectinib. The results showed that the IC_50_ of alectinib in ISK cells was 38.83 μM (logIC_50_=1.589). The IC_50_ toward HEC-1-A cells was 42.70 μM (logIC_50_=1.630). Cell viability decreased in a concentration-dependent manner with increasing alectinib concentration, indicating that alectinib suppresses the proliferation of endometrial cancer cells. These data provided a rational basis for selecting working concentrations in subsequent experiments (**Figure 2A**).

### 3.2 Alectinib increases intracellular PpIX fluorescence

**Figure 3:**
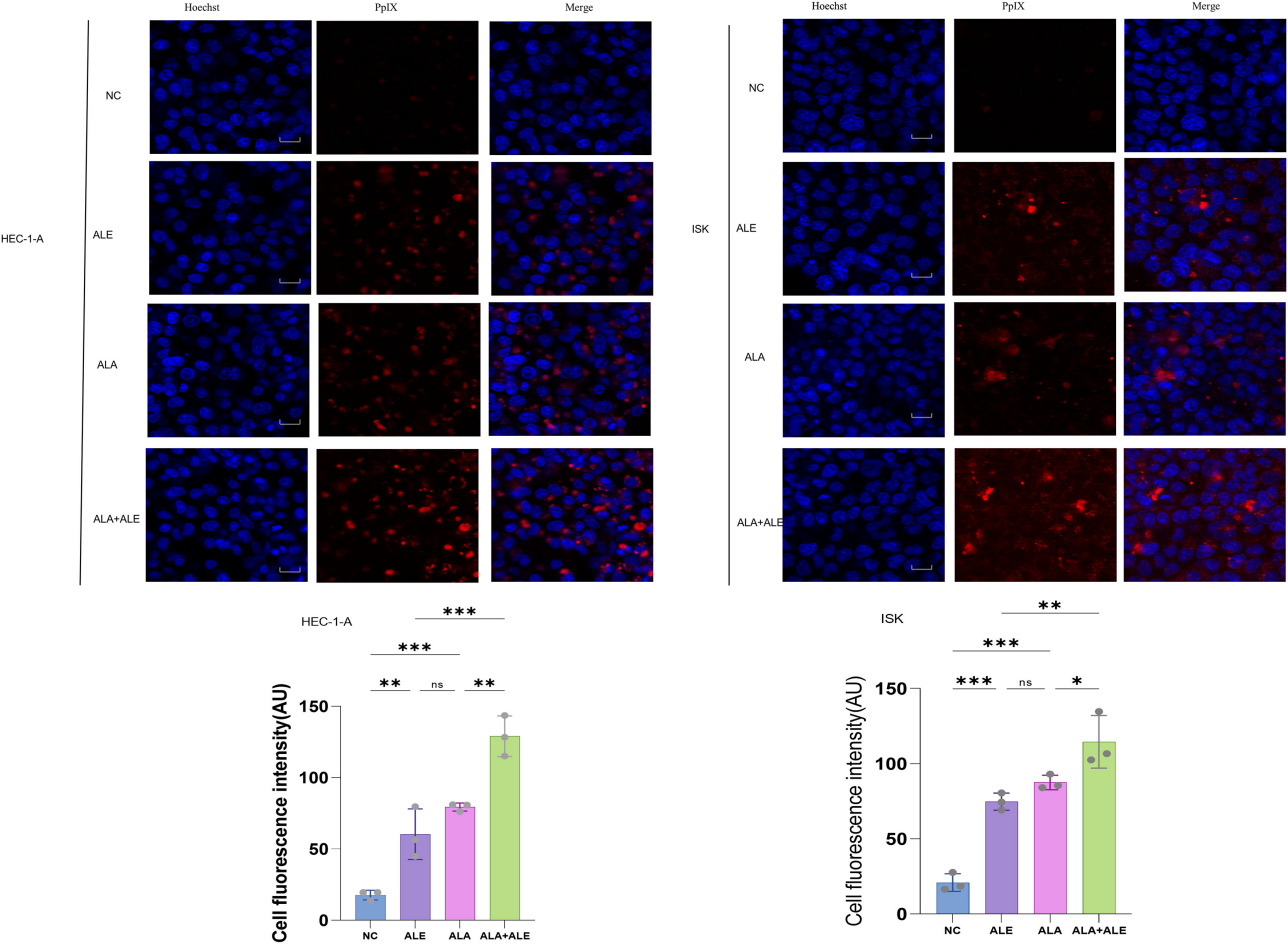
Red fluorescence intensity of HEC-1-A and ISK tumor cells treated with alectinib. *p<0.05, **p<0.01, ***p<0.001; ns=not significant (p≥0.05).

The results of fluorescence signal analysis clearly showed that PpIX was not randomly distributed in the cells, but showed significant subcellular localization specificity. PpIX was mainly enriched and aggregated in the mitochondrial region, and only a weak fluorescence signal or no obvious signal distribution was detected in the nucleus and other parts of the cytoplasm. The target distribution characteristics of PpIX under alectinib were visually reflected (**Figure 2B**). The red fluorescence intensity of HEC-1-A and ISK tumor cells treated with alectinib was significantly higher than that of the control group. After ALA treatment, the red fluorescence intensity of the cells was also enhanced (**Figure 3**).

### 3.3 Alectinib augments ALA-PDT-triggered ROS generation

**Figure 4:**
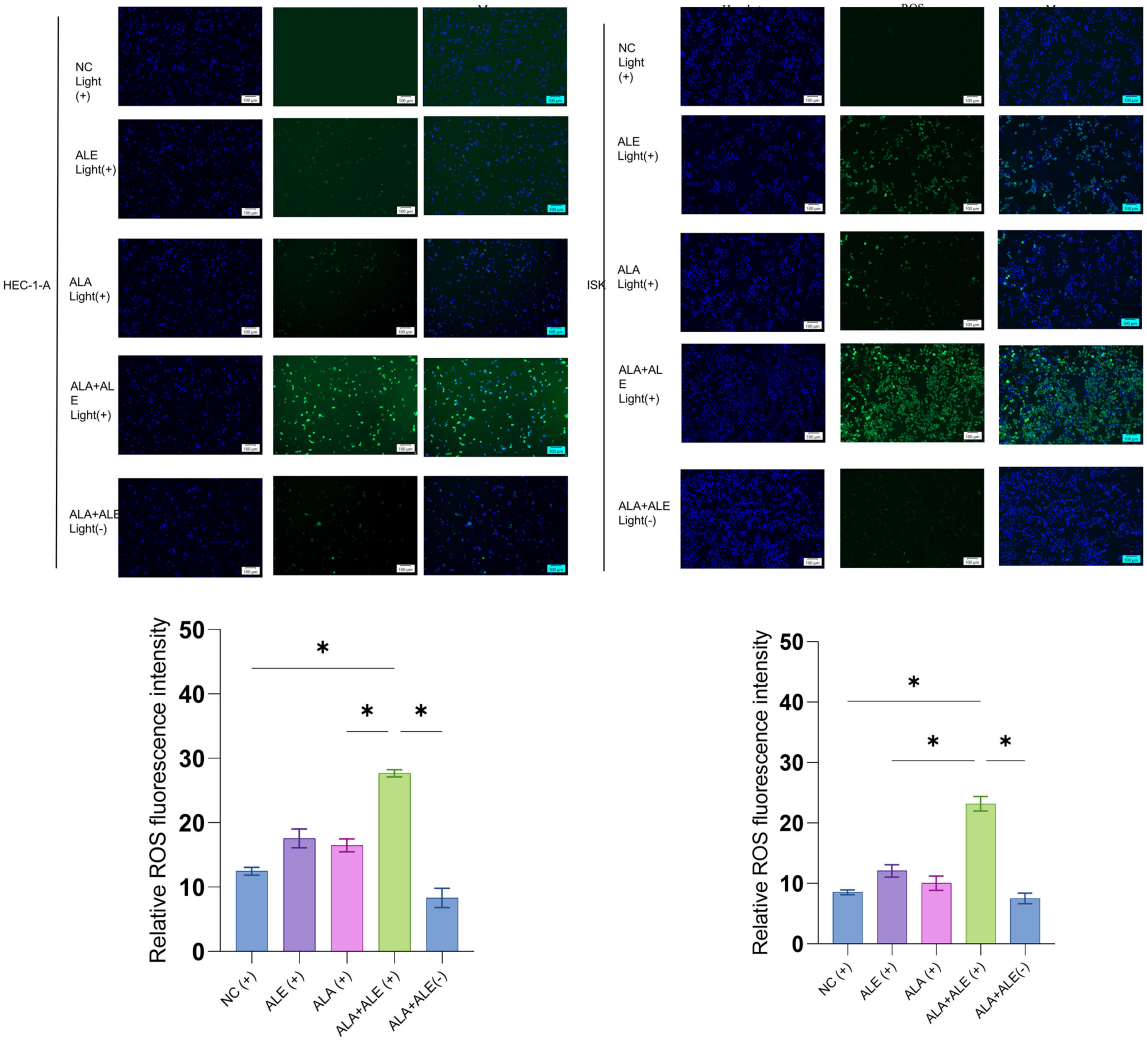
The combination of ALA and ALE induces ROS accumulation in HEC-1-A and ISK cells under light irradiation. *p<0.05.

Intracellular ROS levels were measured by DCFH-DA staining to evaluate the effect of alectinib on ALA-PDT in HEC-1-A and ISK cells. In both cell lines, single treatment with either ALA or alectinib induced only a slight increase in DCF fluorescence. In contrast, combined treatment with 20 μM ALA and 20 μM alectinib under light irradiation resulted in markedly enhanced green fluorescence intensity, indicating robust intracellular ROS accumulation. This increase was significantly higher than that observed in single-agent groups (*P* < 0.05). Notably, the light-negative control group showed no obvious ROS elevation, confirming that ROS production was strictly dependent on LED light irradiation (**Figure 4**).

### 3.4 Alectinib reduces intracellular heme content

**Figure 5:**
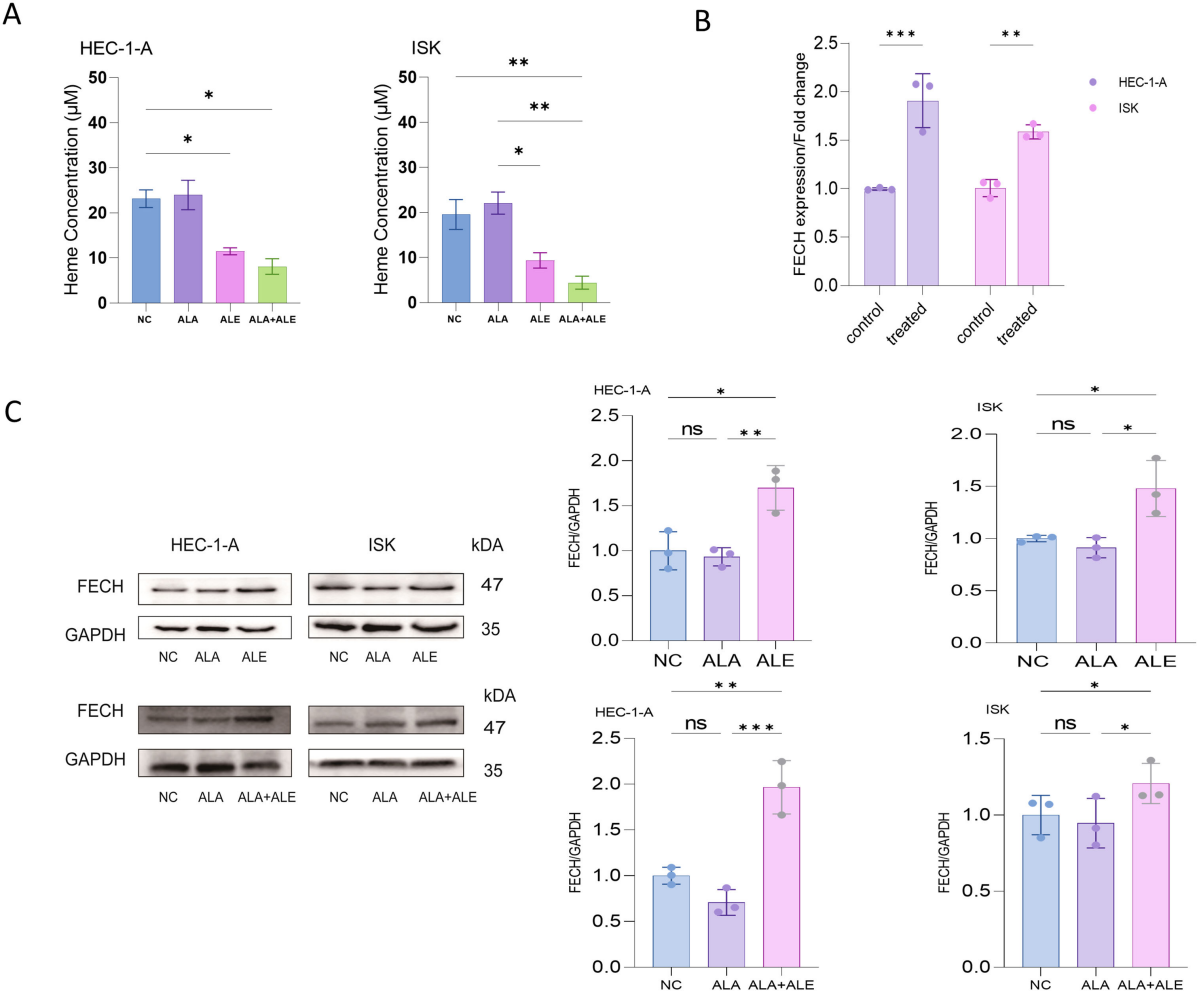
**(A)** Heme concentration decreases in HEC-1-A and ISK cells after treatment with ALE alone or in combination with ALA. **(B)** Alectinib upregulates FECH mRNA expression in endometrial cancer cells. **(C)** Western Blot analysis of FECH protein expression in HEC-1-A and ISK cells treated with ALA/ALE. *p<0.05, **p<0.01, ***p<0.001; ns=not significant (p≥0.05).

To confirm the inhibitory effect of alectinib on *FECH* activity, we measured intracellular heme levels, the terminal product of the heme biosynthesis pathway. In both HEC-1-A and ISK cells, alectinib significantly reduced intracellular heme content compared with the solvent control (P < 0.05). Combination with 20 μM ALA further decreased heme levels, which is consistent with the increased PpIX fluorescence observed in confocal microscopy. The reduction in heme indicated that alectinib suppressed *FECH*-mediated conversion of PpIX to heme, thereby blocking PpIX catabolism and promoting its intracellular accumulation (**Figure 5A**).

### 3.5 Effects of alectinib on *FECH* mRNA expression

After 24 hours of alectinib treatment, qPCR results showed that the expression of *FECH* mRNA was significantly increased in all tumor cell lines. In HEC-1-A cells, the relative expression of *FECH* mRNA in the treatment group was 1.91 ± 0.28 times higher than that in the solvent control group (P < 0.01). In ISK cells, the value was 1.59 ± 0.07 times that of the solvent control group (P < 0.01). This phenomenon suggests that pharmacological inhibition of *FECH* activity by alectinib may activate a negative feedback loop, thereby promoting *FECH* gene transcription (**Figure 5B**).

### 3.6 Effects of alectinib on *FECH* protein expression

To explore the effect of alectinib on the expression of *FECH* itself, we used Western blot analysis to examine *FECH* levels in HEC-1-A and ISK cells after 48 h of treatment. Compared with the negative control (NC) group, *FECH* expression was significantly increased in both the alectinib-only (ALE) group and the ALA plus alectinib (ALA+ALE) group (all P < 0.05). In contrast, *FECH* expression in the ALA-only group showed no significant difference compared with the NC group (P > 0.05) (**Figure 5C**).

### 3.7 Effects of ALA and alectinib on apoptosis of endometrial tumor cells

**Figure 6:**
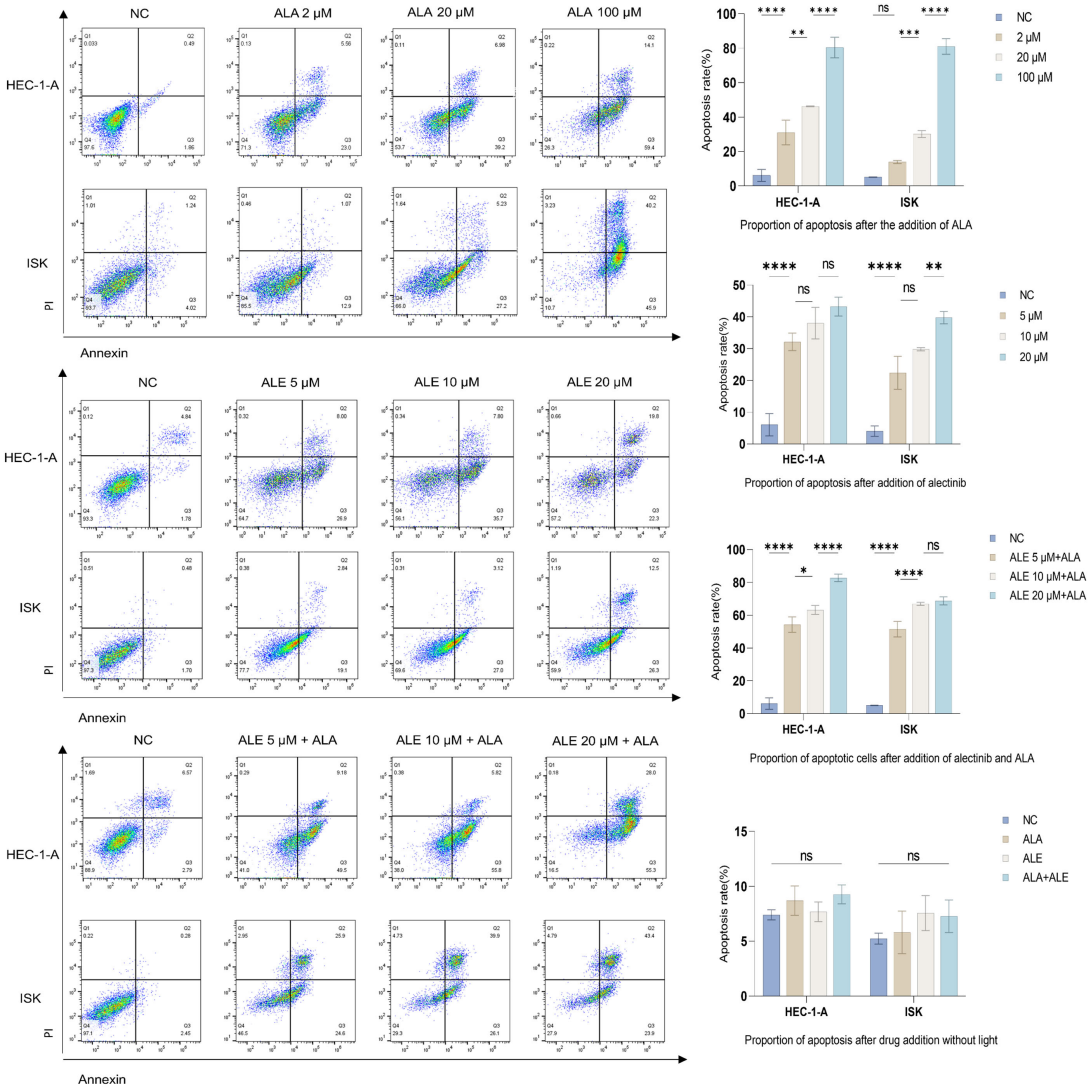
Apoptotic rates of endometrial cancer cells following single or combined treatment with ALA and alectinib. *p<0.05, **p<0.01, ***p<0.001, ****p<0.0001; ns=not significant (p≥0.05).

We first evaluated the apoptotic rate of cells treated with gradient concentrations of ALA, followed by light irradiation (to induce ALA-PDT). In HEC-1-A cells, the apoptosis rates were approximately 28.5% (2 μM ALA), 46.1% (20 μM ALA), and 73.5% (100 μM ALA). To minimize the applied ALA concentration, 20 μM ALA was selected for subsequent combination experiments. We next examined apoptosis after alectinib single treatment. In HEC-1-A cells, the apoptosis rates were 34.9% (5 μM), 43.5% (10 μM), and 42.1% (20 μM). Flow cytometry further showed that combined treatment with 20 μM alectinib and 20 μM ALA achieved a markedly higher apoptotic rate: 83.3% in HEC-1-A cells and 67.3% in ISK cells. These results confirmed the synergistic pro-apoptotic effect of alectinib plus ALA-PDT (**Figure 6**).

## 4 Discussion

This study further confirms that alectinib enhances the accumulation of photosensitizer in tumor cells by targeting *FECH* to block the conversion of PpIX to heme, which extends the “off-target effect” of alectinib (14). Tumor cells are inherently more susceptible to *FECH* inhibition owing to their elevated heme metabolic demand, whereas *FECH* activity in normal tissues remains largely unaffected, thereby limiting nonspecific PpIX accumulation in normal cells (15). Sufficient PpIX accumulation can be achieved without high doses of exogenous ALA, laying a foundation for low-toxicity treatment (16, 17).

The CCK-8 assay revealed that alectinib exerted dose-dependent cytotoxicity against both ISK and HEC-1-A cells, with IC_50_ values of 38.83 μM and 42.70 μM, respectively. These findings are noteworthy for two reasons. First, although EC is not typically classified as an “ALK-driven” malignancy (unlike ALK-positive NSCLC), our results demonstrate that alectinib can still inhibit EC cell proliferation. Previous studies have reported low but detectable ALK expression in ~10–15% of EC tissues, and ALK overexpression has been associated with poor prognosis in EC patients, supporting the potential relevance of ALK as a therapeutic target in this setting (18). Second, the IC_50_ values of alectinib in ISK and HEC-1-A cells were higher than the clinically achievable plasma concentrations at the standard therapeutic dose, suggesting that the antiproliferative effect of alectinib observed *in vitro* may require local accumulation or combined intervention to exert efficacy *in vivo* (19, 20). Previous *in vitro* mechanistic studies have commonly adopted 10 μM alectinib in multiple cell lines, offering a reference concentration range for the development of alectinib-combined photodynamic therapy strategies (21). Notably, the slight difference in IC_50_ between ISK and HEC-1-A cells (38.83 μM vs. 42.70 μM) may reflect inherent biological heterogeneity between these two EC cell lines. These differences are often associated with changes in kinase expression, drug uptake, or DNA repair capacity—all of which can influence drug sensitivity (22). This heterogeneity highlights the need for personalized therapeutic strategies in EC (23).

We observed that alectinib treatment significantly upregulated both mRNA and protein expression of *FECH*, despite reducing intracellular heme levels. This apparent paradox—increased expression accompanied by decreased function—is not necessarily contradictory. We hypothesize that it may reflect a compensatory feedback response: alectinib may inhibit *FECH* catalytic activity (e.g., by interfering with iron insertion or substrate binding), thereby blocking the conversion of PpIX to heme. The consequent depletion of heme could then relieve a heme-dependent transcriptional repression of the *FECH* gene, leading to increased FECH expression. Such a negative feedback loop has been described for other heme biosynthesis enzymes (24). Alectinib may bind to the active site or allosteric pocket of *FECH*, thereby disrupting its ability to interact with PpIX or iron. When the synthesis of heme is blocked, the transcription and translation of the *FECH* gene are activated through negative feedback regulation to compensate for the lack of enzyme activity, which eventually shows the characteristics of “increased expression but decreased activity” (25–27).

In this study, intracellular total heme content was determined, which reliably reflects the alterations of endogenous free heme homeostasis (28). Alectinib-driven *FECH* inhibition lowered total heme levels, disrupted iron homeostasis and mitochondrial function, and attenuated cellular antioxidant capacity. Such heme depletion further sensitized endometrial cancer cells to ALA-PDT-mediated oxidative injury (29). Previous studies have reported the regulatory effect of alectinib on *FECH* activity (13, 30). The present study lacks loss- and gain-of-function validation of *FECH*, and the precise feedback regulatory mechanism remains to be further elucidated. Therefore, future experiments using purified *FECH* for direct activity assays and *FECH*-knockdown or -overexpressing cell lines are required to determine whether alectinib directly inhibits *FECH* enzymatic activity or acts through indirect transcriptional regulation.

The therapeutic potential of the alectinib–ALA combination was further verified by flow cytometry. In ISK cells, combined treatment with ALA and alectinib elevated the apoptosis rate to 67.3%, indicating a prominent combined antitumor effect. This synergy likely stems from two complementary mechanisms. First, as discussed, alectinib enhances PpIX accumulation, which, upon light activation, generates high levels of ROS to trigger oxidative stress-induced apoptosis (31). Second, alectinib itself may induce apoptosis by inhibiting ALK (or off-target kinases), which are critical for activating survival pathways such as PI3K/Akt and Ras/MAPK (32, 33). Blocking these pathways sensitizes EC cells to ROS-mediated cell death, creating a “double hit” that amplifies apoptotic signaling. Our selection of 20 μM as the optimal concentration for both ALA and alectinib in combination experiments was guided by two key considerations. For ALA, concentrations ≥100 μM induced >70% apoptosis alone—excessively high levels that would obscure the improved efficacy of the combination—while 2 μM ALA induced only ~20% apoptosis, providing insufficient signal for combination studies. For alectinib, 20 μM was below the IC_50_ (38.83 μM for ISK cells), ensuring that the enhanced apoptotic response was not due to the additive cytotoxicity of high-dose alectinib alone. Notably, the “light-positive” vs. “light-negative” controls confirmed that PpIX-mediated apoptosis is strictly light-dependent—consistent with the mechanism of PDT. This control is critical, as it rules out the possibility that PpIX itself (without light activation) contributes to the observed apoptosis, confirming that the improved therapeutic outcome originates from alectinib-promoted PpIX accumulation coupled with light-triggered ROS generation. The present study was limited to *in vitro* endometrial cancer cell models, and further *in vivo* experimental validation is warranted in future investigations.

In conclusion, alectinib enhances ALA-PDT efficacy, laying a theoretical foundation for novel conservative therapeutic strategies for endometrial cancer and warranting further clinical translational investigation.

## Data Availability

The original contributions presented in the study are included in the article/supplementary material. Further inquiries can be directed to the corresponding authors.
